# Prediction of *Oncomelania hupensis* distribution in association with climate change using machine learning models

**DOI:** 10.1186/s13071-023-05952-5

**Published:** 2023-10-23

**Authors:** Ning Xu, Yun Zhang, Chunhong Du, Jing Song, Junhui Huang, Yanfeng Gong, Honglin Jiang, Yixin Tong, Jiangfan Yin, Jiamin Wang, Feng Jiang, Yue Chen, Qingwu Jiang, Yi Dong, Yibiao Zhou

**Affiliations:** 1 Fudan University School of Public Health, Shanghai, 200032 China; 2 Key Laboratory of Public Health Safety, Fudan University, Ministry of Education, Shanghai, 200032 China; 3 Fudan University Center for Tropical Disease Research, Shanghai, 200032 China; 4 Yunnan Institute of Endemic Disease Control and Prevention, Dali, 671000 Yunnan China; 5 Yunnan Provincial Key Laboratory of Natural Focal Disease Prevention and Control Technology, Dali, 671000 Yunnan China; 6 School of Epidemiology and Public Health, Faculty of Medicine, University of Ottawa, Ottawa, Canada

**Keywords:** Schistosomiasis, *Oncomelania hupensis*, Species distribution, Ecological niche model, Climate change

## Abstract

**Background:**

*Oncomelania hupensis* is the sole intermediate host of *Schistosoma japonicum*. Its emergence and recurrence pose a constant challenge to the elimination of schistosomiasis in China. It is important to accurately predict the snail distribution for schistosomiasis prevention and control.

**Methods:**

Data describing the distribution of *O. hupensis* in 2016 was obtained from the Yunnan Institute of Endemic Disease Control and Prevention. Eight machine learning algorithms, including eXtreme Gradient Boosting (XGB), support vector machine (SVM), random forest (RF), generalized boosting model (GBM), neural network (NN), classification and regression trees (CART), k-nearest neighbors (KNN), and generalized additive model (GAM), were employed to explore the impacts of climatic, geographical, and socioeconomic variables on the distribution of suitable areas for *O. hupensis.* Predictions of the distribution of suitable areas for *O. hupensis* were made for various periods (2030s, 2050s, and 2070s) under different climate scenarios (SSP126, SSP245, SSP370, and SSP585).

**Results:**

The RF model exhibited the best performance (AUC: 0.991, sensitivity: 0.982, specificity: 0.995, kappa: 0.942) and the CART model performed the worst (AUC: 0.884, sensitivity: 0.922, specificity: 0.943, kappa: 0.829). Based on the RF model, the top six important variables were as follows: Bio15 (precipitation seasonality) (33.6%), average annual precipitation (25.2%), Bio2 (mean diurnal temperature range) (21.7%), Bio19 (precipitation of the coldest quarter) (14.5%), population density (13.5%), and night light index (11.1%). The results demonstrated that the overall suitable habitats for *O. hupensis* were predominantly distributed in the schistosomiasis-endemic areas located in northwestern Yunnan Province under the current climate situation and were predicted to expand north- and westward due to climate change.

**Conclusions:**

This study showed that the prediction of the current distribution of *O. hupensis* corresponded well with the actual records. Furthermore, our study provided compelling evidence that the geographical distribution of snails was projected to expand toward the north and west of Yunnan Province in the coming decades, indicating that the distribution of snails is driven by climate factors. Our findings will be of great significance for formulating effective strategies for snail control.

**Graphical Abstract:**

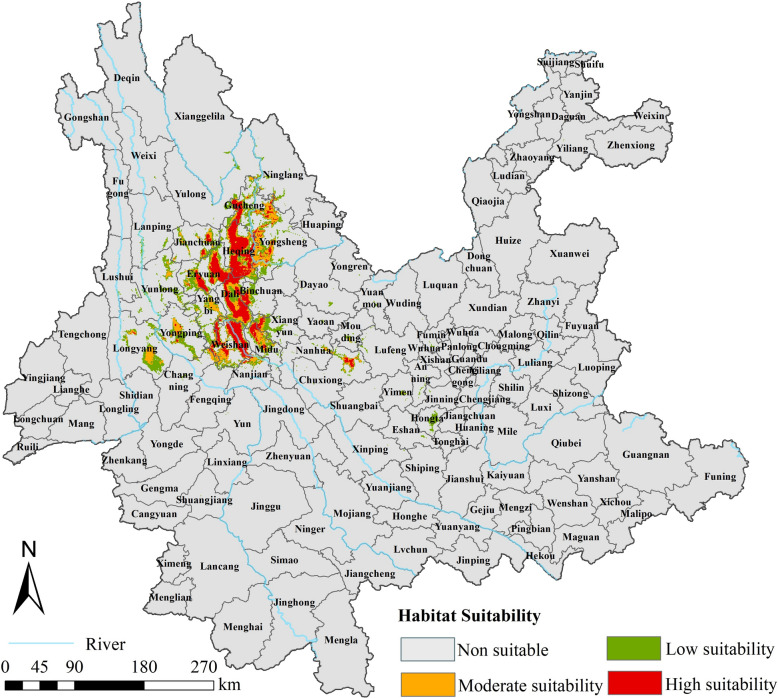

**Supplementary Information:**

The online version contains supplementary material available at 10.1186/s13071-023-05952-5.

## Background

Schistosomiasis, a neglected tropical disease, is prevalent worldwide, particularly in areas with poor public health, afflicting more than 250 million people [1]. In China, schistosomiasis, caused by *Schistosoma japonicum*, has mainly affected 12 provinces along the Yangtze River and is considered a major public health concern [2]. After more than 70 years of national integrative prevention, schistosomiasis in China is currently under control with a low level of prevalence [3, 4]. However, new and recurring breeding sites of *Oncomelania hupensis,* the only known intermediate host of *S. japonicum* [5], are constantly emerging, posing a serious challenge to the elimination of schistosomiasis. In 2021, a total of 1063 hm^2^ of emerging snail habitats and 5113 hm^2^ of re-emerging snail habitats were reported [6]. Under the guidance of the Healthy China 2030 initiative and the Healthy China Action Plan (2019–2030) [7], China is approaching schistosomiasis elimination. A means for accurate prediction of the distribution of *O. hupensis* would greatly facilitate its progress [8].

Ecological niche models predict which areas meet the ecological requirements of a species by analyzing the geographical distribution of the target species and linking it to environmental factors within the location [9]. They have been successfully used in predicting species distribution, risk assessment of invasive alien species, conservation of endangered species, and mapping the risk of disease transmission [10–12].

Considering that the geographical distribution of *O. hupensis* overlaps with areas where schistosomiasis is endemic [13], understanding the relationship between snail breeding sites and their corresponding environmental determinants is important for accurately determining the distribution of snails and is ultimately crucial to interrupting the spread of schistosomiasis. Previously, studies have been conducted to predict the potential distribution of snails in China; however, large-scale predictions are no longer sufficient to meet the requirements for fine control of snails at this stage [14, 15]. Studies have shown that factors influencing species distribution may vary at different scales, leading to differences in distribution ranges and even producing opposite conclusions [16, 17]. In addition, there are three types of schistosomiasis-endemic areas in China according to the geographical environment and the epidemiological pattern of schistosomiasis: (1) marshland and lake regions, (2) mountainous and hilly regions, and (3) water network regions [18]. The main environmental factors that influence the distribution of snails vary in different schistosomiasis-endemic areas [19]. There are also many subspecies or geographical strains of snails in mainland China, and each one may have a different ecology and may be influenced by climate change differently [20]. In addition, the local agricultural structure is closely related to the distribution of snails [21]. As the proportion of paddy fields increases, the probability of snail habitats also increases [22]. Also, irrigation canals or ditches play a significant role in the reproduction of snails. During irrigation, snails can spread through the water flow in the channels and survive in suitable environments [23, 24].

Yunnan was once one of the provinces with a high prevalence of schistosomiasis due to its unique geographical location [25]. The schistosomiasis in Yunnan Province has been effectively controlled since 2009 through the implementation of comprehensive strategies [26]. By the end of 2022, seven of the 18 endemic counties/districts/cities in Yunnan Province had met the transmission interruption criteria and 11 counties/districts/cities had met the elimination criteria [27]. However, the complex natural environment in the endemic areas makes it difficult to further compress the snail’s breeding areas, and the cost of controlling residual snails increases considerably [28]. Moreover, the use of molluscicides has been hampered by the restoration of wetlands in ecological reserves, which provides a suitable breeding environment for snails. It is difficult to monitor snails through conventional approaches in these sites, leading to an underestimation of the distribution of snails and increasing the risk of schistosomiasis transmission [21, 25, 29].

Machine learning algorithms have been increasingly applied to model ecological niches [30]. Using various machine learning methods, our study aims to investigate the determinants for *O. hupensis* occurrence and predict the distribution of suitable areas for *O. hupensis* under different climate scenarios*.* The results of the present study will provide a theoretical basis for the fine control of *O. hupensis*.

## Methods

### Study area

Yunnan Province is a hilly/mountainous schistosomiasis-endemic area in southwestern China, with a subtropical and tropical monsoon climate. The province is bordered by Myanmar to the west and Laos and Vietnam to the south and southeast, respectively, with the Lancang, Nu, Jinsha, Lidu, Yuan, and Nanpan rivers flowing through the province. The mild climate, abundant water resources, and dense vegetation provide favorable natural conditions for the survival of snails.

### Distribution records for *O. hupensis*

**Fig. 1:**
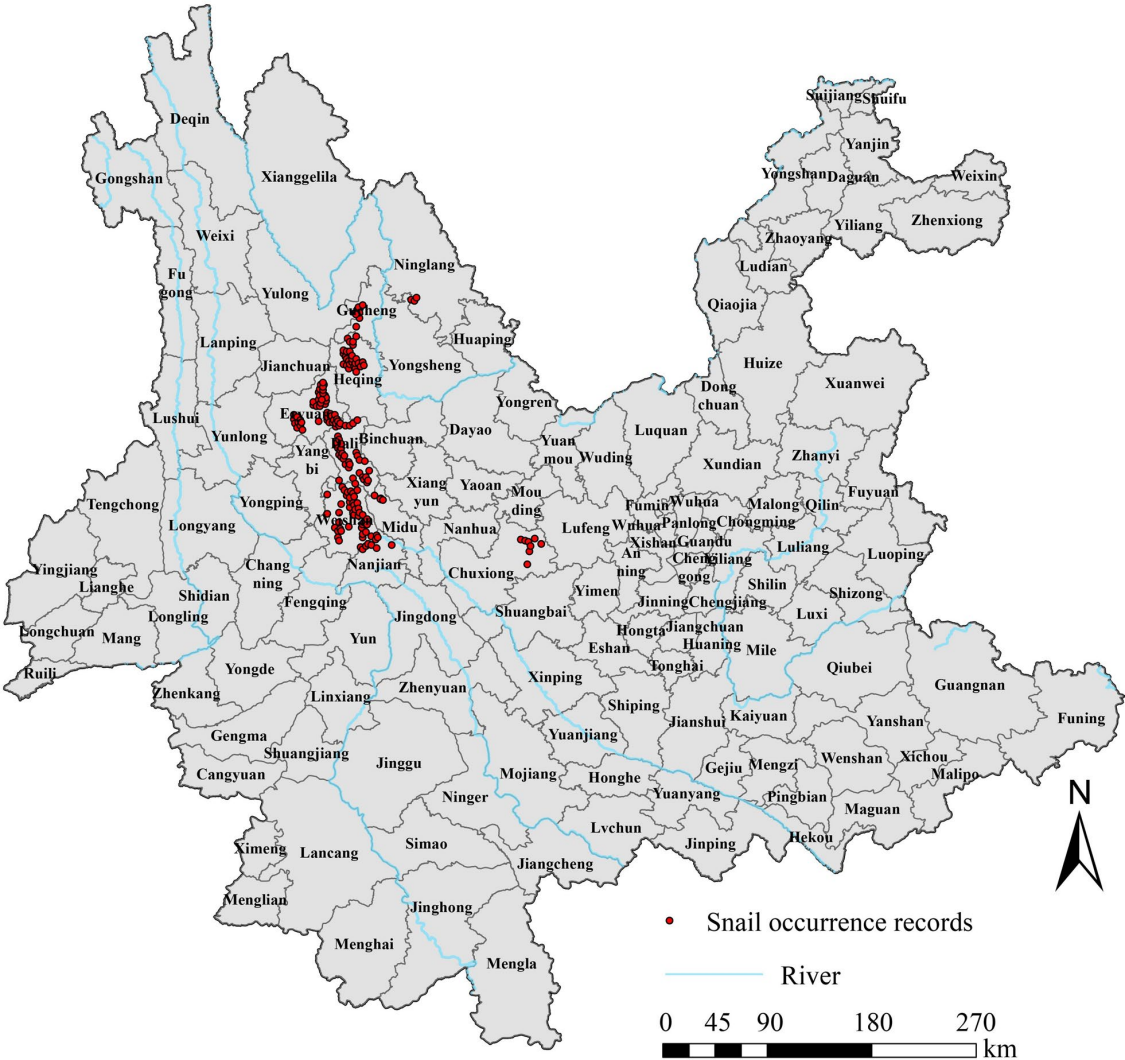
Distribution of *O. hupensis* in Yunnan Province in 2016

Distribution records for *O. hupensis*, including longitude and latitude, were obtained from the survey of *O. hupensis* conducted by the Yunnan Institute of Endemic Disease Control and Prevention in 2016. The survey utilized systematic sampling methods in conjunction with environmental sampling techniques. Sites of *O. hupensis* presence were found in Gucheng District, Heqing County, Ninglang County, Yulong County, Eryuan County, Dali City, Weishan County, Nanjian County, Midu County, and Chuxiong City (Fig. 1). To avoid spatial autocorrelation, we first filtered the data by removing multiple records that appeared in the same grid (resolution of 1 km × 1 km) and keeping only one record [31]. Finally, 184 presence records were retained, and absence sites were generated in the study area at a ratio of 1:2 for constructing the model.

### Environmental variables

The distribution of *O. hupensis* is influenced by multiple factors [14, 32]. In this study, climatic, geographical, and socioeconomic factors in Yunnan Province were obtained from various public datasets. Among the climate-related variables, average annual precipitation (AAP), average annual temperature (AAT), annual accumulated temperature ≥ 0 °C (AAT0), annual accumulated temperature ≥ 10 °C (AAT10), aridity (AR), and moisture index (IM) were obtained from the Chinese Academy of Sciences, Resource and Environmental Sciences and Data Center (http://www.resdc.cn/). The remaining 19 bioclimatic variables (current period) calculated on the basis of monthly temperature and precipitation values from 1970 to 2000 [33], were downloaded from the WorldClim website (https://www.worldclim.org/), with a spatial resolution of 1 km × 1 km. The future bioclimatic variables modeled by the Beijing Climate Center-Climate System Model version 2-Middle Resolution (BCC-CSM2-MR), which is better able to simulate temperature changes in China [34], were also obtained from the WorldClim website, with the same spatial resolution. The present study included four sets of emission scenarios (Shared Socioeconomic Pathways [SSPs]) classified by CO_2_ emissions, namely low (SSP126), medium (SSP245), medium-high (SSP370), and high (SSP585), for three periods comprising the 2030s, 2050s, and 2070s [35].

Geographical factors included slope, elevation (EL), normalized difference vegetation index (NDVI), and data on the distance to waterways (DST) that are typically depicted and labeled in the OpenStreetMap (OSM) using specific “waterway” tags, including large rivers, canals, lakes, and other important water bodies. Socioeconomic factors included human footprint (HFP), night light index (NLI), population density (DP), and gross domestic product (GDP). HFP is an indicator of human footprint activity, with values ranging from 0 to 50, where a value of zero represents “natural” areas with no human activity, and values above 20 correspond to areas with intense human activity [36]. These data were downloaded from the Chinese Academy of Sciences, Resources and Environmental Sciences Data Center (http://www.resdc.cn/), Socioeconomic Data and Applications Center (http://sedac.ciesin.columbia.edu) and the WorldPop website (http://www.worldpop.org). All environmental data were in raster format and resampled to the same spatial resolution (1 km × 1 km) and then cropped to the Yunnan Province region using ArcGIS 10.4.

To avoid multicollinearity of the environmental variables, correlation analysis was conducted in R 4.2.1, and variables with absolute values of correlation coefficients ≥ 0.85 were considered highly correlated. We utilized the following criteria to select the most predictive variable: in datasets of the same type of variables, such as climatic, geographical, or socioeconomic factors, variables related to most variables and with more biological significance are retained for model construction, while other related variables are deleted [37, 38].

### Ecological niche modeling

Eight machine learning algorithms in the Caret package, namely, eXtreme Gradient Boosting (XGB), support vector machine (SVM), random forest (RF), generalized boosted model (GBM), neural network (NN), classification and regression trees (CART), k-nearest neighbors (KNN), and generalized additive model (GAM), were utilized to predict the suitable distribution of snails. The original datasets were randomly divided into two parts, with 70% of the datasets used as training samples for model construction and the remaining 30% labeled as testing samples for evaluating the accuracy of the models. For different models, the optimal hyperparameters, which were set to control the behavior of the learning algorithm, were determined using the grid search method and 10-fold cross-validation, such as the *mtry* in the RF model, and the predictive power of the model was tested using the testing dataset to select a model with the best predictive performance.

The final output of the prediction model represents the probability of snail presence, ranging from 0 to 1. We define areas with a presence probability of less than 0.40 as non-suitable areas, 0.41–0.60 as areas with low suitability, 0.61–0.80 as areas with moderate suitability, and greater than 0.80 as areas with high suitability [39]. ArcGIS 10.4 was applied to classify the different levels of areas.

### Model evaluations

Common model evaluation metrics include the area under the receiver operating characteristic curve (AUC), sensitivity, specificity, and kappa [19, 40]. The AUC value is the most common evaluation indicator for ecological niche models, and the closer the value is to 1, the higher the accuracy of the model. The sensitivity indicates the predictive accuracy for presence. The specificity implies the predictive accuracy for absence. Kappa ranges from –1 to 1, with a value closer to 1 indicating that the predicted results are consistent with the actual observations.

## Results

### Variable selection

**Fig. 2:**
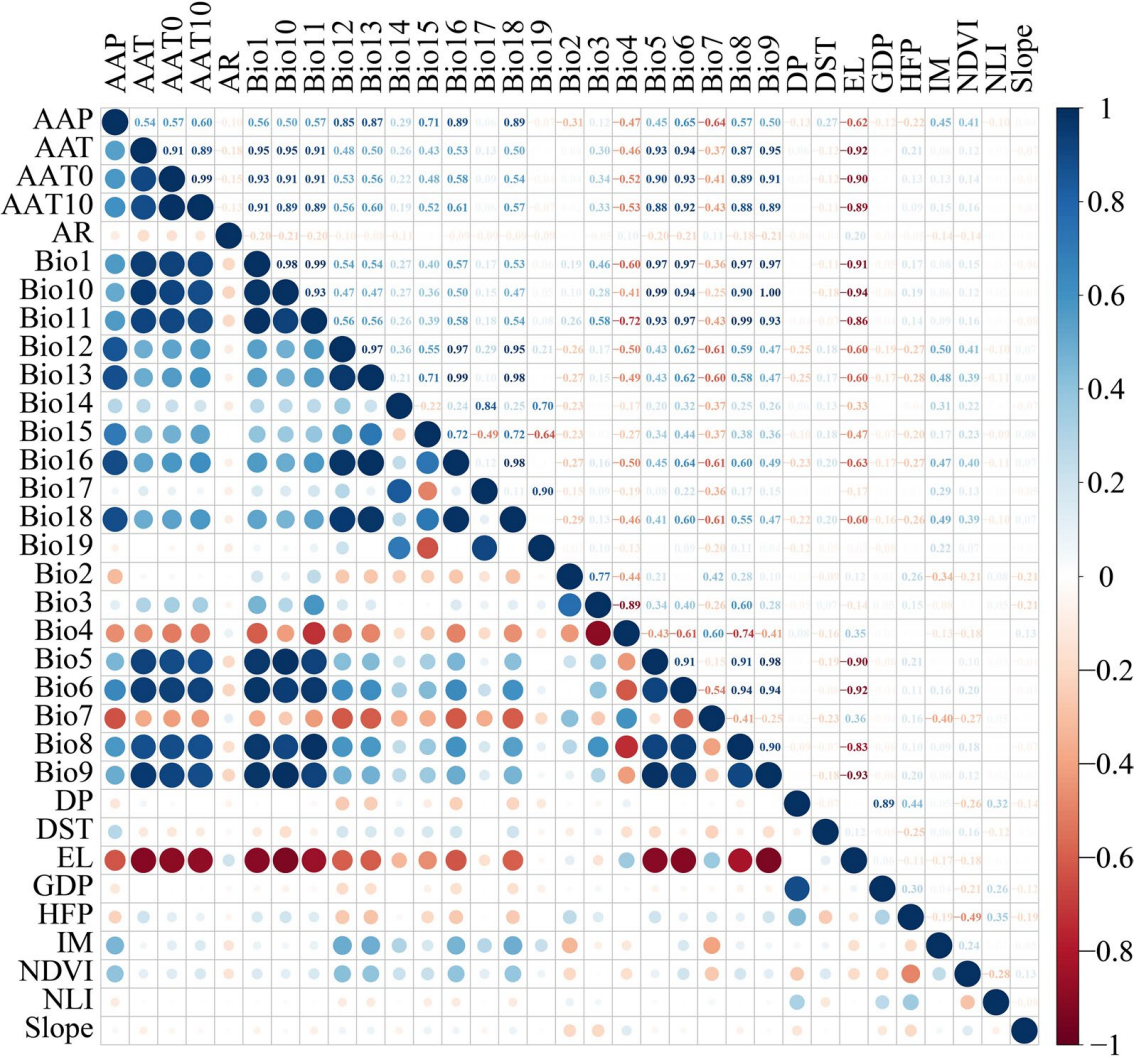
Correlation analysis of variables. Blue and red indicate the strength of positive and negative correlations, respectively. AAP, average annual precipitation; AAT, average annual temperature; AAT0, annual accumulated temperature ≥ 0 °C; AAT10, annual accumulated temperature ≥ 10 °C; AR, aridity; DP, population density; DST, distance to the waterway; EL, elevation; GDP, gross domestic product; HFP, human footprint; IM, moisture index; NDVI, normalized difference vegetation index; NLI, night light index

Figure 2 illustrates that most variables exhibited strong correlations. Specifically, for climatic factors, Bio9 exhibited a strong correlation with AAT, AAT0, AAT10, Bio1, Bio5, Bio6, Bio8, Bio10, and Bio11; thus Bio9, which also correlated with EL, was retained as a predictive variable. Similarly, AAP correlated with Bio12, Bio13, Bio16, and Bio18. Therefore, AAP was retained as a predictive variable. Due to the strong correlation between Bio17 and Bio19, the latter was retained as a predictive variable. There was a strong correlation between Bio3 and Bio4, and Bio4 was retained for model construction due to its significant contribution to predicting the potential habitats of snails [41]. For socioeconomic factors, DP, which demonstrates a powerful predictive capability for the distribution of snails [42], was correlated with GDP, and hence DP was retained as a predictive variable. Finally, 16 variables were employed in the model development process, including 10 climatic variables (Bio2, Bio4, Bio7, Bio9, Bio14, Bio15, Bio19, AR, AAP, and IM), three geographical variables (slope, NDVI, and DST), and three socioeconomic variables (HFP, DP, and NLI) (Table 1).

**Table 1:** Variables involved in model building

Classification	Variables	Units	Description	Years	Sources
Climatic	Bio2	°C	Mean diurnal temperature range	1970–2000	https://www.worldclim.org/
	Bio4	–	Temperature seasonality		
	Bio7	°C	Temperature annual range		
	Bio9	°C	Mean temperature of the driest quarter		
	Bio14	mm	Precipitation of the driest month		
	Bio15	–	Precipitation seasonality (coefficient of variation)		
	Bio19	mm	Precipitation of the coldest quarter		
	AR	–	Aridity	1950–1990	http://www.resdc.cn/
	AAP	mm	Average annual precipitation	2015	
	IM	%	Moisture index	1950–1990	
Geographical	Slope	–	Slope from dem	2000	http://www.resdc.cn/
	NDVI	–	Normalized difference vegetation index	2015	
	DST	km	Distance to waterway	2016	http://www.worldpop.org
Socioeconomic	HFP	–	Human footprint	1993–2009	http://sedac.ciesin.columbia.edu
	NLI	–	Night light index	2015	http://www.resdc.cn/
	DP	persons/km^2^	Population density	2015	http://www.worldpop.org

### Model performance

Table 2 illustrates the predictive performance of the models employed, including AUC, sensitivity, specificity, and kappa. Among the eight models, the RF model exhibited the best prediction performance (AUC: 0.991 [95% CI 0.989–0.993]; sensitivity: 0.982; specificity: 0.995; kappa: 0.942), followed by the GBM (AUC: 0.983 [95% CI 0.973–0.993], sensitivity: 0.981; specificity: 0.991; kappa: 0.932). The CART model performed the worst (AUC: 0.884 [95% CI 0.863–0.905]); sensitivity: 0.922; specificity: 0.943; kappa: 0.829). Therefore, we applied the optimized RF model to predict the distribution of suitable areas for *O. hupensis* under current and future climate conditions.

**Table 2:** Comparison of the prediction performance of different models

Model	AUC (95% CI)	Sensitivity	Specificity	Kappa
RF	0.991 (0.989–0.993)	0.982	0.995	0.942
GBM	0.983 (0.973–0.993)	0.981	0.991	0.932
XGB	0.980 (0.966–0.994)	0.962	0.945	0.889
KNN	0.979 (0.962–0.996)	0.975	0.849	0.789
GAM	0.973 (0.946–0.999)	0.868	0.962	0.842
NN	0.969 (0.932–0.987)	0.959	0.940	0.917
SVM	0.896 (0.874–0.920)	0.679	0.981	0.724
CART	0.884 (0.863–0.905)	0.922	0.943	0.829

RF, random forest; GBM, generalized boosted model; XGB, eXtreme Gradient Boosting; KNN, k-nearest neighbors; GAM, generalized additive model; NN, neural network; SVM, support vector machine; CART, classification and regression trees

### Importance of variables

**Fig. 3:**
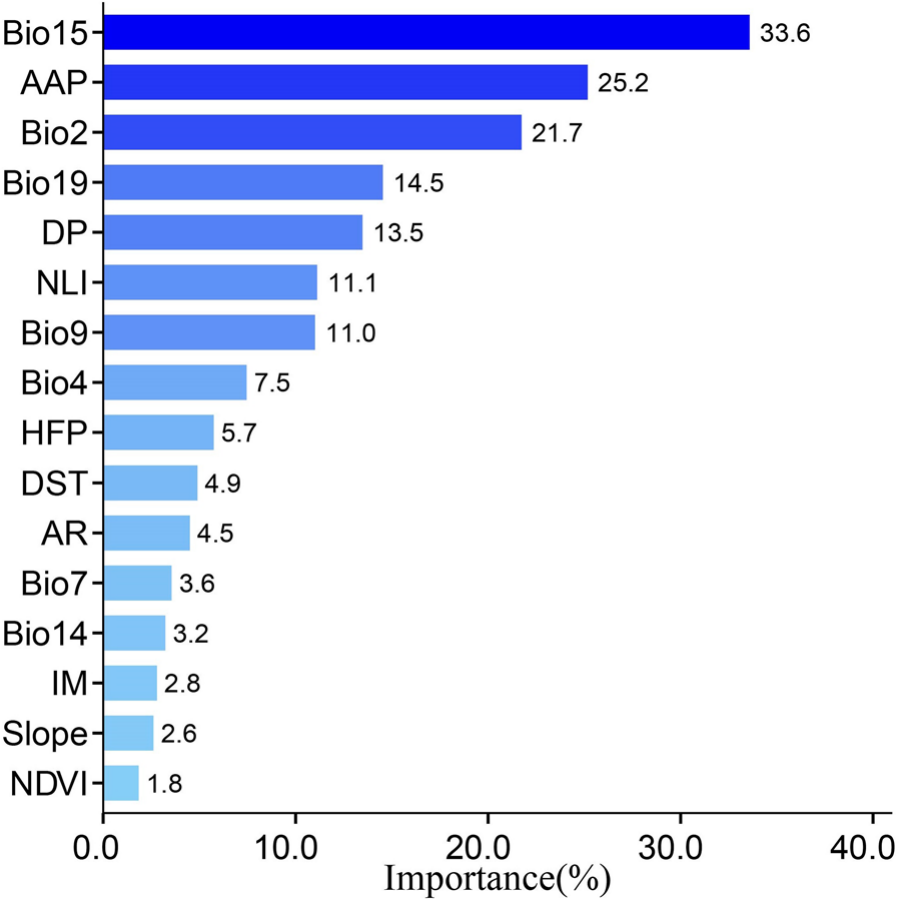
Importance of variables in the random forest model. AAP, average annual precipitation; DP, population density; NLI, night light index; HFP, human footprint; DST, distance to the waterway; AR, aridity; IM, moisture index; NDVI, normalized difference vegetation index

Figure 3 illustrates the importance of the variables measured using MeanDecreaseGini, with higher values indicating greater importance in the model. From the RF model, the top six important variables were Bio15 (33.6%), AAP (25.2%), Bio2 (21.7%), Bio19 (14.5%), DP (13.5%), and NLI (11.1%). Among those six factors, four were climatic factors, and the remaining two were related to socioeconomic conditions, indicating that climatic factors were the main factors determining the distribution of *O. hupensis.*

### The current distribution of suitable areas for *O. hupensis* in Yunnan Province

**Fig. 4:**
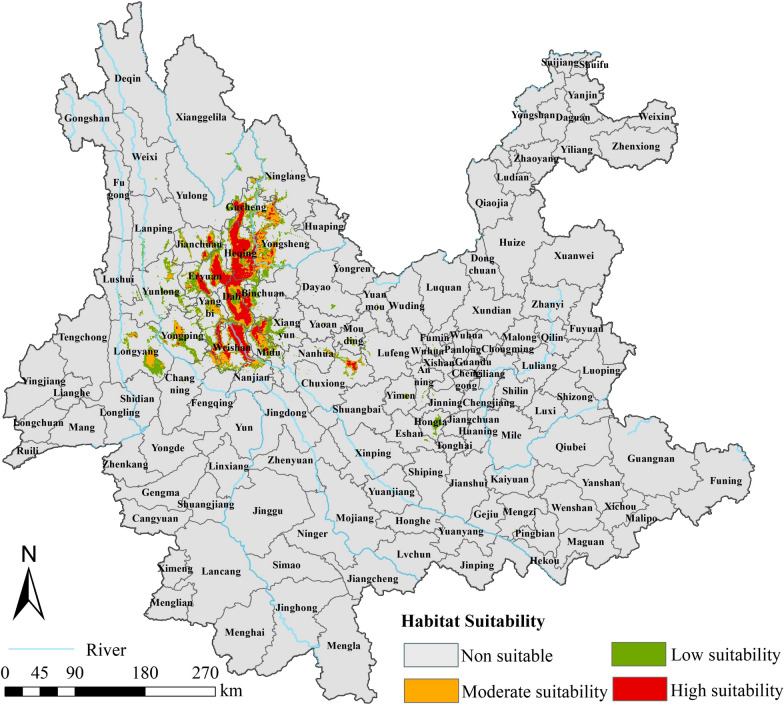
The current distribution of suitable areas for *O. hupensis* in Yunnan Province predicted with the random forest model

The established optimal RF model was utilized to predict the spatial distribution of suitable areas for *O. hupensis* in Yunnan Province (Fig. 4). The prediction results showed that non-suitable, low-suitability, moderate-suitability, and high-suitability areas accounted for 96.0%, 1.8%, 1.1%, and 1.1%, respectively. The overall suitable areas that deserve high priority for monitoring were predominantly distributed in the schistosomiasis-endemic areas located in northwestern Yunnan Province, including Gucheng District, Heqing County, Dali City, Weishan County, Midu County, western Yongsheng County, northwestern Binchuan County, eastern Jianchuan County, western Xiangyun County, northern Nanjian County, and northeastern Chuxiong City, with the high-suitability areas mainly in Heqing County, Eryuan County, Dali City, and Weishan County. Areas with moderate and low suitability were distributed around areas with high suitability. It is noteworthy that in Longyang District and Yongping County, which are adjacent to schistosomiasis-endemic areas of Yunlong County and Yangbi County, snails have never been found previously. However, the predictions in this study indicated the existence of suitable habitats for snails in these two locations, cautioning against the potential spread of snails from schistosomiasis-endemic areas to non-endemic areas.

### Changes in suitable areas for *O. hupensis* in the coming decades in Yunnan Province

**Fig. 5:**
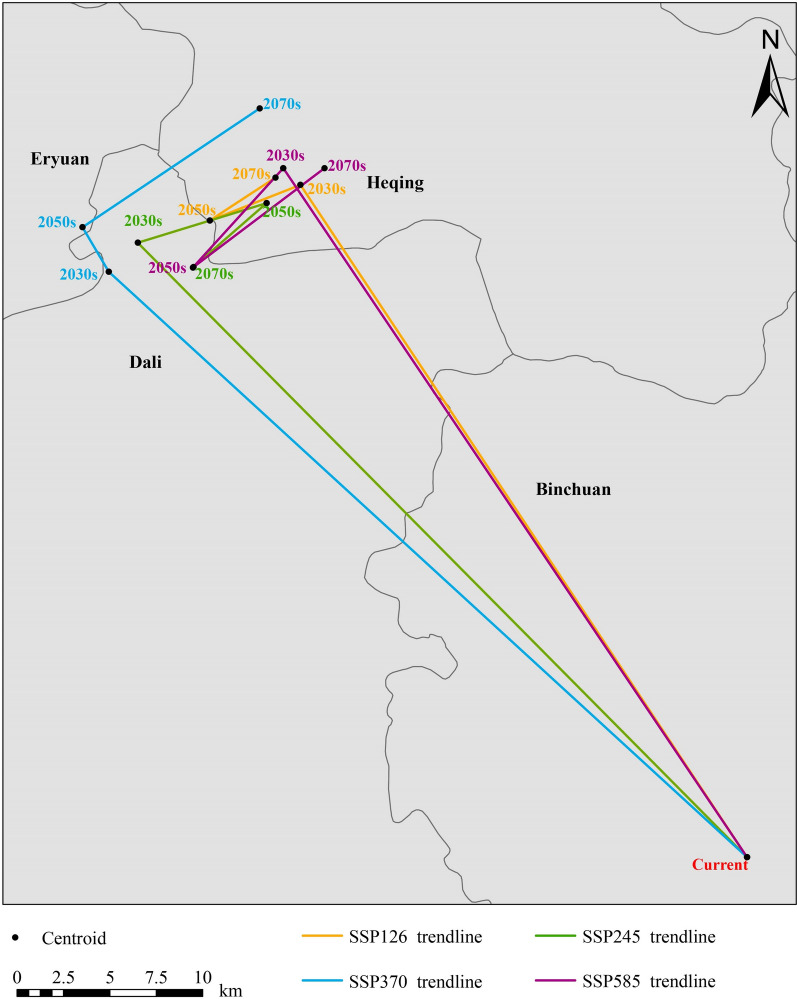
The shifts of the suitable areas centroid for *O. hupensis*

Figure 5 shows that under current conditions, the centroid of suitable areas for *O. hupensis* was in Binchuan County, Yunnan Province, located at 100.46°E, 25.73°N. Overall, under the future climate scenarios (SSP126, SSP 245, SSP 370, and SSP 585) for the 2030s, 2050s, and 2070s, the centroid of suitable areas was expected to shift northwest, primarily located in Dali City, Heqing County, and Eryuan County. Specifically, under the SSP126 scenario, it was projected that in the 2030s, the centroid of suitable areas would shift to Heqing County at 100.25°E, 26.06°N, and then move to 100.20°E, 26.04°N in the 2050s.

Subsequently, in the 2070s, it was predicted to be located at 100.24°E, 26.06°N. Under the SSP245 scenario, the centroid of suitable areas was anticipated to move to Dali City at 100.17°E, 26.03°N in the 2030s, and shift to Heqing County at 100.23°E, 26.05°N in the 2050s. Subsequently, it was projected to move to Dali City at 100.20°E, 26.02°N in the 2070s. Under the SSP370 scenario, it was estimated that in the 2030s, the centroid of suitable areas would shift to Dali City at 100.16°E, 26.02°N, and move to Eryuan County at 100.14°E, 26.04°N in the 2050s. In the 2070s, a shift to Heqing County at 100.23°E, 26.10°N was projected. Under the SSP585 scenario, the centroid of suitable areas was expected to move to Heqing County at 100.24°E, 26.07°N in the 2030s and shift to Dali City at 100.20°E, 26.02°N in the 2050s. Then, in the 2070s, another migration to Heqing County, positioned at 100.26°E, 26.07°N, was projected.

By the 2030s and 2050s, the suitable areas would cover almost the whole of Gucheng District, Heqing County, Jianchuan County, Eryuan County, Dali City, and Yangbi County under the future climate scenarios (Additional file 1: Fig. S1 and Additional file 2: Fig. S2). On the contrary, suitable areas in southern Weishan County were projected to have shrunk significantly. By the 2070s under SSP126 and SSP370, the suitable areas in Weishan County would have been substantially reduced, retaining a small part of suitable areas in the north. The suitable areas in Chuxiong City would remain generally unchanged over time.

**Fig. 6:**
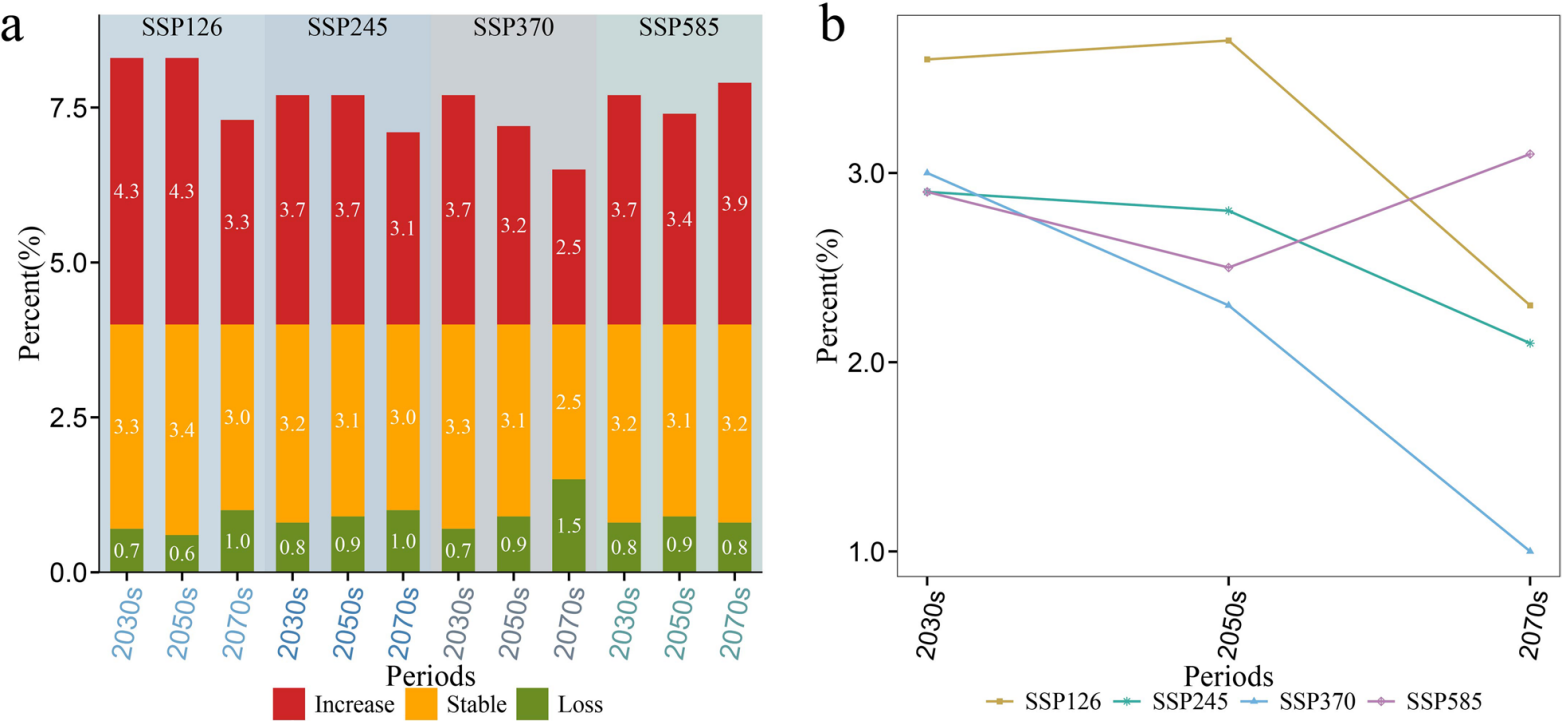
The changes in the percentage of increase, loss, and stable suitable areas (**a**) and the net increase in the percentage of suitable areas (net increase = increase − loss) (**b**) for *O. hupensis* between the current time and the periods of the 2030s, 2050s, and 2070s under different climate scenarios (SSP126, SSP245, SSP370, and SSP585) in Yunnan Province. Increase, newly suitable areas; Stable: unaltered suitable areas; Loss: loss of suitable areas

Figure 6a demonstrates that, in the future climate scenario, an additional 2.5%–4.3% of the areas would become suitable for *O. hupensis*, while 0.6%–1.5% of the original suitable areas would no longer be climatically favorable, with the largest additional suitable areas and the smallest reduction in the 2050s under SSP126. Compared to suitable areas for *O. hupensis* under the current condition, the net increase of suitable areas would exceed 2% under future climate scenarios, except for the 2070s in SSP370. In addition, the net increase in suitable areas under future scenarios would experience a gradual decline from the 2030s to the 2070s, except for SSP585 (Fig. 6b).

## Discussion

Machine learning models can better handle multidimensional data and have been widely used for parasitic disease risk prediction and vector spread trends [43, 44]. Previous research has shown that presence/absence-based models were superior to presence-based models in terms of predictive performance [19]. In this study, we employed eight presence/absence-based machine learning models, and the RF model showed the best predictability. Similarly, in previous studies on the distribution of snails, the RF model outperformed other models based on AUC evaluation metrics [39, 42]. The RF model is an ensemble learning method based on the automatic combination of a set of tree-like predictors and is able to resist overfitting its training set to a certain extent [45]. However, Zheng found that the XGB model, originally introduced in 2016 [46], had better predictability based on the same metrics [47]. Differences in both the size of the sample and the environmental variables incorporated can lead to discrepancies in model prediction performance [48, 49], which may explain the performance variation in different models across studies. In addition to optimizing the model, the accuracy of model prediction could be improved by including appropriate factors related to the distribution of the species.

Climatic factors played an important role in the distribution of *O. hupensis*. Of the top four significant climatic variables, three were related to precipitation, and one was related to temperature. This is supported by the physiological characteristics of *O. hupensis* that it prefers regions with appropriate warmth and humidity. From a precipitation perspective, Bio15, representing precipitation seasonality (coefficient of variation), had a major impact on the survival of *O. hupensis*. As an amphibious snail, *O. hupensis* requires water for development during its juvenile stage and is more likely to grow in moist soil during its adult stage, which receives its moisture principally from precipitation. With respect to the temperature-related variable, Bio2, representing the mean diurnal temperature range, contributed more significantly to shaping the geographical distribution of *O. hupensis* than other temperature-related variables. As a narrowly temperate mollusk, its growth and development can be influenced by temperature through the regulation of enzyme activity and expression of related genes [32]. In addition, DP and NLI also greatly impacted the distribution of snails, with a cumulative importance of over 20.0%. Human population dynamics are often considered to be a major contributor to altering the natural environment. In addition, humans can also directly bring snails to other areas for dispersal, such as the construction of flood control embankments, transportation of seeds, and ditch irrigation [50, 51]. The NLI has been applied to assess ecological status, as it reflects the level of urbanization and acts as an indicator of human activity [52–54]. Also, a previous study showed that the NLI was positively correlated with the concentrations of contaminants in the soil, which may alter the microenvironment where snails live [53].

Based on the outputs of the prediction model, we divided Yunnan Province into non-suitable areas and areas with low, moderate, or high suitability for *O. hupensis* to determine the hot spots for snail control. The suitable areas for snail survival under the current climate situation were primarily in the northwestern Yunnan Province, which basically coincided with the actual presence records of *O. hupensis* as well as the predictions of the risk areas for schistosomiasis transmission reported by Hu et al.[55], indicating that our prediction model was scientific and reasonable. Heqing County had the largest suitable area in our study. Consistently, a snail survey conducted in 2021 found that Changtou village of Heqing County ranked first in terms of recurrence of *O. hupensis* among the 32 villages investigated in 18 endemic counties [56]. Meanwhile, it is worth noting that our prediction model also found additional suitable regions where *O. hupensis* has so far been unrecorded but may require further investigation, including Yongping County and Longyang District, due to the possibility of human activities introducing snails into areas suitable for their survival, thus facilitating their dispersal [50, 51].

Theoretically, climate change drives shifts in the geographical range of species, resulting in migration to areas with climatically suitable habitats [57]. Similar to the results from a previous study [15], our model showed that the suitable areas in Yunnan Province tended to expand in the north and shrink in the south with respect to the current distribution. The southern part of the province is expected to have higher temperatures and lower precipitation in the future, which would discourage the survival of snails [58]. However, in contrast to the prediction based on national data that most parts of Yunnan Province would be suitable for *O. hupensis* in the future [15], our results revealed that the suitable areas were more concentrated in certain counties/districts/cities, which would need to be a focus for targeted surveys. More accurate distribution maps were provided based on our fine-scale projections, which enabled the health authorities to specify and optimize targeted snail control strategies.

Some counties in Yunnan Province, such as Binchuan, Jianchuan, Yangbi, and Yunlong, have already met the schistosomiasis elimination standard, and the snail habitats have vanished. However, our research predicted that these counties would continue harboring favorable habitats for snails in the long term. As a result, continuous monitoring is necessary. Inadequate control measures could facilitate the spread of snails and increase the risk of schistosomiasis transmission. Previous studies have reported a resurgence of live snails in Guangdong Province 27 years after they were last found, possibly due to the incomplete monitoring and eradication of snails as well as the environmental conditions suitable for snails created by flooding in recent years [59]. A resurgence of snails has also been reported in Chuxiong, Yunnan Province [60]. Moreover, human activities such as ditch irrigation could introduce snails to suitable habitats, resulting in cross-watershed spread [50]. Hence, the suitable areas are a focus for control and should be given high priority.

This study had certain limitations. Firstly, snail control measures were not considered in the predictive model. The inclusion of snail control measures, such as pharmaceutical measures and environmental modifications, may improve model prediction. Secondly, no external validation was performed to assess the predictive accuracy of the model. However, according to the snail survey in Yunnan Province in 2019, snails were detected mainly in the counties/cities where the high-suitability areas for *O. hupensis* were located in the prediction map, such as Heqing County, Eryuan County, Dali City, and Weishan County, with over 10 hm^2^ of snail habitats in each county/city [61]. Moreover, the snail survey in Yunnan Province in 2021 showed that, among the 32 villages surveyed in the 18 endemic counties /districts/cities, Changtou village in Heqing County ranked first in terms of areas of *O. hupensis* recurrence, which was consistent with our prediction that Heqing County had the largest suitable areas for snails [56].

## Conclusions

In conclusion, the RF model demonstrated the best performance in predicting the distribution of suitable areas for snails. Suitable areas were predominantly distributed in the northwestern part of Yunnan Province under the current climate condition and would expand north- and westward. Small-scale predictions were more precise in identifying the habitats of snails and could then offer finer guidance for the control of snails. Our findings also suggested that areas that had met schistosomiasis elimination criteria were still ecologically suitable for snail growth and thus more rigorous surveillance should be carried out in these areas to prevent the recurrence of snails.

### Supplementary Information

**Additional file 1: Figure S1**. Distribution of suitable areas for *O. hupensis* in the 2030s, 2050s, and 2070s under the SSP126 and SSP245 climate scenarios predicted with the random forest model. Increase, newly suitable areas; Stable: unaltered suitable areas; Loss: loss of suitable areas.

**Additional file 2: Figure S2**. Distribution of suitable areas for *O. hupensis* in the 2030s, 2050s, and 2070s under the SSP370 and SSP585 climate scenarios predicted with the random forest model. Increase, newly suitable areas; Stable: unaltered suitable areas; Loss: loss of suitable areas.

## Data Availability

The datasets used and/or analyzed during the current study are available from the corresponding author upon reasonable request.

## Abbreviations

AAP: Average annual precipitation
AAT: Average annual temperature
AAT0: Annual accumulated temperature ≥ 0 °C
AAT10: Annual accumulated temperature ≥ 10 °C
AR: Aridity
AUC: Area under the receiver operating characteristic curve
BCC-CSM2-MR: Beijing Climate Center-Climate System Model version 2-Middle Resolution
CART: Classification and regression trees
DP: Population density
DST: Distance to the waterway
EL: Elevation
GAM: Generalized additive model
GBM: Generalized boosted model
GDP: Gross domestic product
HFP: Human footprint
IM: Moisture index
KNN: K-nearest neighbors
NDVI: Normalized difference vegetation index
NLI: Night light index
NN: Neural network
RF: Random forest
SVM: Support vector machine
XGB: eXtreme Gradient Boosting

## References

[1] Barnett R (2018). Schistosomiasis. Lancet.

[2] Lv S, Tian LG, Liu Q, Qian MB, Fu Q, Steinmann P, Chen J-X, Yang G-J, Yang K, Zhou X-N (2013). Water-related parasitic diseases in China. Int J Environ Res Public Health.

[3] Wang L, Utzinger J, Zhou XN (2008). Schistosomiasis control: experiences and lessons from China. Lancet.

[4] Guo JY, Xu J, Zhang LJ, Lv S, Cao CL, Li SZ, Zhou XN (2020). Surveillance on schistosomiasis in five provincial-level administrative divisions of the People’s Republic of China in the post-elimination era. Infect Dis Poverty.

[5] Utzinger J, Zhou XN, Chen MG, Bergquist R (2005). Conquering schistosomiasis in China: the long march. Acta Trop.

[6] Zhang LJ, Xu ZM, Yang F, He JY, Dang H, Li YL, Cao CL, Xu J, Li SZ, Zhou XN (2022). Progress of schistosomiasis control in People’s Republic of China in 2021. Chin J Schisto Control..

[7] Xu J, Lv S, Cao CL, Li SZ, Zhou XN (2018). Progress and challenges of schistosomiasis elimination in China. Chin J Schisto Control.

[8] Wang W, Yang K (2020). Implementation of precision control to facilitate the progress towards schistosomiasis elimination in China. China Trop Med.

[9] Sillero N (2011). What does ecological modeling model? A proposed classification of ecological niche models based on their underlying methods. Ecol Model.

[10] Johnson EE, Escobar LE, Zambrana-Torrelio C (2019). An ecological framework for modeling the geography of disease transmission. Trends Ecol Evol.

[11] Elith J, Leathwick JR (2009). Species distribution models: ecological explanation and prediction across space and time. Annu Rev Ecol Evol S.

[12] Hulagappa T, Baradevanal G, Surpur S, Raghavendra D, Doddachowdappa S, Shashank PR, Mallaiah KK, Bedar J (2022). Diagnosis and potential invasion risk of *Thrips parvispinus* under current and future climate change scenarios. PeerJ.

[13] Yang X, Zhang Y, Sun QX, Zhou JX, Zhou XN (2019). SWOT analysis on snail control measures applied in the national schistosomiasis control programme in the People’s Republic of China. Infect Dis Poverty.

[14] Gong YF, Li YL, Zhang LJ, Lv S, Xu J, Li S (2021). The potential distribution prediction of *Oncomelania hupensis* based on newly emerging and reemergent habitats-China, 2015–2019. China CDC Wkly.

[15] Gong YF, Hu XK, Hao YW, Luo ZW, Feng JX, Xue JB, Guo ZY, Li YL, Zhang LJ, Xia S, Shan LY (2022). Projecting the proliferation risk of *Oncomelania hupensis* in China driven by SSPs: a multi-scenario comparison and integrated modeling study. Adv Clim Change Res..

[16] Wang RL, Jiang CX, Guo X, Chen DD, You C, Zhang Y, Wang M, Li Q (2020). Potential distribution of *Spodoptera frugiperda* (JE Smith) in China and the major factors influencing distribution. Glob Ecol Conserv..

[17] Sun X, Long ZX, Jia JB (2022). Identifying core habitats and corridors for giant pandas by combining multiscale random forest and connectivity analysis. Ecol Evol.

[18] Zou L, Ruan S (2015). Schistosomiasis transmission and control in China. Acta Trop.

[19] Zhang J, Yue M, Hu Y, Bergquist R, Su C, Gao F, Cao ZG, Zhang Z (2020). Risk prediction of two types of potential snail habitats in Anhui Province of China: model-based approaches. PLoS Negl Trop Dis.

[20] Zhu GP, Fan JY, Peterson AT (2017). *Schistosoma japonicum* transmission risk maps at present and under climate change in mainland China. PLoS Negl Trop Dis.

[21] Yang K, Wang XH, Yang GJ, Wu XH, Qi YL, Li HJ, Zhou XN (2008). An integrated approach to identify distribution of *Oncomelania hupensis*, the intermediate host of *Schistosoma japonicum*, in a mountainous region in China. Int J Parasitol.

[22] Qiu J, Li RD, Xu XJ, Yu CH, Xia X, Hong XC, Chang B, Yi F, Shi Y (2014). Identifying determinants of *Oncomelania hupensis* habitats and assessing the effects of environmental control strategies in the plain regions with the waterway network of China at the microscale. Int J Environ Res Public Health.

[23] Shi YY, Qiu J, Li RD, Shen Q, Huang D (2017). Identification of potential high-risk habitats within the transmission reach of *Oncomelania hupensis* after floods based on SAR techniques in a plane region in China. Int J Environ Res Public Health.

[24] Yang Y, Zhou YB, Song XX, Li SZ, Zhong B, Wang TP, Bergquist R, Zhou XN, Jiang QW (2016). Integrated control strategy of Schistosomiasis in the People’s Republic of China: projects involving agriculture, water conservancy, forestry, sanitation and environmental modification. Adv Parasitol.

[25] Song J, Shen MF, Dong Y (2022). The effect analysis of comprehensive governance for schistosomiasis in Yunnan Province from 2004 to 2021. J Trop Dis Parasitol.

[26] Hao Y, Zheng H, Zhu R, Guo JG, Wang LY, Chen Z, Zhou X (2010). Schistosomiasis situation in People’s Republic of China in 2009. Chin J Schisto Control..

[27] Zhang LJ, He JY, Yang F, Dang H, Li YL, Guo SY, Li S, Cao C, Xu J, Li S, Zhou X (2023). Progress of schistosomiasis control in People’s Republic of China in 2022. Chin J Schisto Control..

[28] Song J, Dong Y, Shen MF, Xiong MT, Zhang Y, Wang LF, Chen C, Sun J, Du C (2021). Analysis of the risk assessment result of schistosomiasis transmission in Yunnan Province in 2020. Chin J Schisto Control..

[29] Shen MF, Feng XG, Huang NB, Zhang Y, Wu MS, Song J, Xiong MT, Wang LF (2016). Analysis of *Oncomelania hupensis* status in schistosomiasis surveillance sites of Yunnan Province in 2015. Chin J Schisto Control.

[30] Zhang J, Li S. A Review of machine learning based species’ distribution modelling. In 2017 International conference on industrial informatics-computing technology, intelligent technology, industrial information integration (ICIICII), Wuhan, China; 2017. p. 199–206.

[31] Echeverry-Cardenas E, Lopez-Castaneda C, Carvajal-Castro JD, Aguirre-Obando OA (2021). Potential geographic distribution of the tiger mosquito *Aedes albopictus* (Skuse, 1894) (Diptera: Culicidae) in current and future conditions for Colombia. PLoS Negl Trop Dis.

[32] Liu MM, Feng Y, Yang K (2021). Impact of micro-environmental factors on survival, reproduction and distribution of *Oncomelania hupensis* snails. Infect Dis Poverty.

[33] Fick SE, Hijmans RJ (2017). WorldClim 2: new 1-km spatial resolution climate surfaces for global land areas. Int J Climatol.

[34] Wu TW, Song LC, Li WP, Wang ZZ, Zhang H, Xin XG, Zhang Y, Zhang L, Li J, Wu F, Liu Y (2014). An overview of BCC climate system model development and application for climate change studies. J Meteorol Res..

[35] Zhang JM, Peng XY, Song ML, Li ZJ, Xu XQ, Wang W (2022). Effects of climate change on the distribution of wild *Akebia trifoliata*. Ecol Evol.

[36] Venter O, Sanderson EW, Magrach A, Allan JR, Beher J, Jones KR, Possingham HP, Laurance WF, Wood P, Fekete BM, Levy MA (2016). Global terrestrial human footprint maps for 1993 and 2009. Sci Data.

[37] Xue JB, Hu XK, Hao YW, Gong YF, Wang XY, Huang LY, Lv S, Xu J, Li S, Xia S (2023). Transmission risk predicting for Schistosomiasis in mainland China by exploring ensemble ecological niche modeling. Trop Med Infect Dis..

[38] Ruan GJ, Li XY, Yuan F, Cammarano D, Ata-UI-Karim ST, Liu XJ, Tian Y, Zhu Y, Cao W, Cao Q (2022). Improving wheat yield prediction integrating proximal sensing and weather data with machine learning. Comput Electron Agric.

[39] Gong YF, Luo ZW, Feng JX, Xue JB, Guo ZY, Jin YJ, Yu Q, Xia S, Lü S, Xu J, Li S (2022). Prediction of trends for fine⁃scale spread of *Oncomelania hupensis* in Shanghai Municipality based on supervised machine learning models. Chin J Schisto Control..

[40] Shabani F, Kumar L, Ahmadi M (2018). Assessing accuracy methods of species distribution models: AUC, specificity, sensitivity and the true skill statistic. Glob J Hum Soc Sci.

[41] Shi Y (2021). Environmental factors shaping spatial distribution of schistosome-transmitting snail *Oncomelania hupensis* and prediction of potential habitats in Dongting lake basin.

[42] Shi QW, Gong YF, Zhao J, Qin ZQ, Zhang J, Wu JZ, Hu Z, Li S (2022). Spatial and temporal distribution pattern of *Oncomelania hupensis* caused by multiple environmental factors using ecological niche models. Front Environ Sci.

[43] Tong YX, Xia ZG, Wang QY, Xu N, Jiang HL, Wang ZZ, Xiong Y, Yin JF, Huang JH, Jiang F, Chen Y (2023). Prediction of the risk distributions for *Anopheles sinensis*, a vector for malaria in Shanghai, China. Am J Trop Med Hyg.

[44] Cenni L, Simoncini A, Massetti L, Rizzoli A, Hauffe HC, Massolo A (2023). Current and future distribution of a parasite with complex life cycle under global change scenarios:* Echinococcus multiloculari*s in Europe. Glob Change Biol.

[45] Breiman L (2001). Random forests. Mach Learn.

[46] Sheridan RP, Wang WM, Liaw A, Ma JS, Gifford EM (2016). Extreme gradient boosting as a method for quantitative structure–activity relationships. J Chem Inf Model.

[47] Zheng JX. Prediction on transmission risk of schistosomiasis and liver flukes diseases in China and Mekong River Basin. Chinese Centre for Disease Control and Prevention; 2021.

[48] McPherson JM, Walter J, Rogers DJ (2004). The effects of species’ range sizes on the accuracy of distribution models: ecological phenomenon or statistical artefact?. J Appl Ecol.

[49] Beauregard F, de Blois S (2014). Beyond a climate-centric view of plant distribution: edaphic variables add value to distribution models. PLoS ONE.

[50] Chen S, Lu D, Duan L, Ma B, Lv C, Li YL, Lu SN, Li LH, Xu L, Wu ZS, Xia S (2022). Cross-watershed distribution pattern challenging the elimination of *Oncomelania hupensis*, the intermediate host of *Schistosoma japonica*, in Sichuan province, China. Parasit Vectors.

[51] Zhang L (2016). Human behavior’s influence on the transmission of Schistosomiasis around Dongting Lake. J Sci Technol.

[52] Ma T, Zhou CH, Pei T, Haynie S, Fan JF (2012). Quantitative estimation of urbanization dynamics using time series of DMSP/OLS nighttime light data: a comparative case study from China’s cities. Remote Sens Environ.

[53] Feng SS, Lu HW, Tian PP, Xue YX, Lu JZ, Tang M, Feng W (2020). Analysis of microplastics in a remote region of the Tibetan Plateau: implications for natural environmental response to human activities. Sci Total Environ.

[54] Shi ZY, Wang YT, Zhao Q (2023). Analysis of spatiotemporal changes of ecological environment quality and its coupling coordination with urbanization in the Yangtze River Delta Urban Agglomeration, China. Int J Environ Res Public Health.

[55] Hu XK, Hao YW, Xia S, Guo YH, Xue JB, Zhang Y, Wang L, Dong Y, Xu J, Li S (2020). Detection of schistosomiasis transmission risks in Yunnan Province based on ecological niche modeling. Chin J Parasitol Parasit Dis..

[56] Shen MF, Du CH, Song J, Wang LF, Sun JY, Chen CQ, Feng X, Zhang Z, Jiang H, Zhou J, Dong Y (2023). The risk surveillance of schistosomiasis in Yunnan, 2021. Chin J Schisto Control..

[57] Chen IC, Hill JK, Ohlemuller R, Roy DB, Thomas CD (2011). Rapid range shifts of species associated with high levels of climate warming. Science.

[58] Gao D, Xie M, Chen X, Wang TJ, Zhan CC, Ren JY, Liu Q (2019). Modeling the effects of climate change on surface ozone during summer in the Yangtze River Delta Region, China. Int J Environ Res Public Health.

[59] Huang SY, Mao Q, Zhong QL, Fan XH, Li WQ, Rao YH, Pei F, Li S, Deng Z (2021). Reappearance of risk of Schistosomiasis transmission and the response after 27 years of interrupted transmission-Guangdong Province, China, 2019. China CDC Wkly.

[60] Wang JX (2013). *Oncomelania* snail recurrence after schistosomiasis transmission interrupted in Chuxiong City. Chin J Schisto Control.

[61] Yunnan Institute for Endemic Disease Control and Prevention (2019). Map of the distribution and prevalence of schistosomiasis in Yunnan Province.

